# Supplementary figures and images for: flowEMMi: an automated model-based clustering tool for microbial cytometric data
Source: BMC Bioinformatics. 2019 Dec 9;20:643. doi: 10.1186/s12859-019-3152-3 (PMC6902487; doi:10.1186/s12859-019-3152-3)

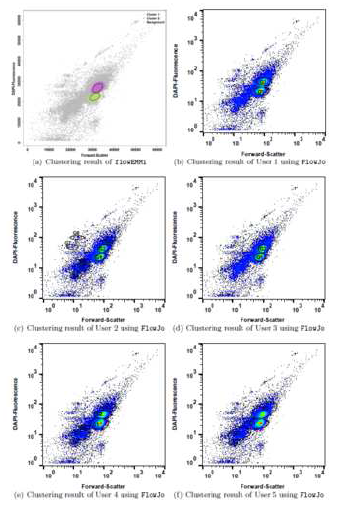

Supplement: Supplementary file 1 — Additional file 1 Clustering results for sample InTH_160719_039 using flowEMMi with 2 congruent cell clusters and 94.1% foreground cells (a) and manual clustering performed by 5 expert users using FlowJo (b-f). User 1 selected 2 cell clusters with 89.9% foreground cells (b). User 2 selected 8 cell clusters with 93.4% foreground cells (c). User 3 selected 2 cell clusters with 91.1% foreground cells (d). User 4 selected 6 cell clusters with 98.6% foreground cells (e). User 5 selected 4 cell clusters with 97.7% foreground cells (f). The label of the clusters selected by using FlowJo is in accordance with the colours of the clusters calculated by flowEMMi. The mean values and abundances of all cell clusters calculated by flowEMMi and FlowJo can be found in the additional file 039.csv. [file 12859_2019_3152_MOESM1_ESM.png]

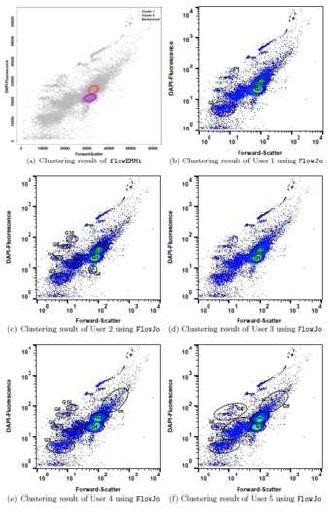

Supplement: Supplementary file 2 — Additional file 2 Clustering results for sample InTH_160728_034 using flowEMMi with 2 congruent cell clusters and 94.1% foreground cells (a) and manual clustering performed by 5 expert users using FlowJo (b-f). User 1 selected 3 cell clusters with 88.8% foreground cells (b). User 2 selected 10 cell clusters with 94% foreground cells (c). User 3 selected 2 cell clusters with 88.7% foreground cells (d). User 4 selected 9 cell clusters with 99% foreground cells (e). User 5 selected 7 cell clusters with 100% foreground cells (f). The label of the clusters selected by using FlowJo is in accordance with the colours of the clusters calculated by flowEMMi. The mean values and abundances of all cell clusters calculated by flowEMMi and FlowJo can be found in the additional file 034.csv. [file 12859_2019_3152_MOESM2_ESM.png]

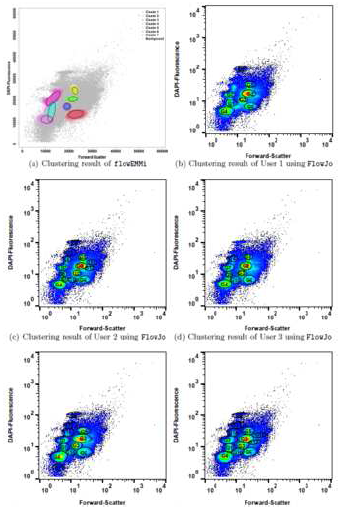

Supplement: Supplementary file 3 — Additional file 3 Clustering results for sample InTH_160720_026 using flowEMMi with 7 congruent cell clusters and 76.4% foreground cells (a) and manual clustering performed by 5 expert users using FlowJo (b-f). User 1 selected 8 cell clusters with 76% foreground cells (b). User 2 selected 14 cell clusters with 82.8% foreground cells (c). User 3 selected 9 cell clusters with 79.5% foreground cells (d). User 4 selected 12 cell clusters with 86.9% foreground cells (e). User 5 selected 13 cell clusters with 95.9% foreground cells (f). The label of the clusters selected by using FlowJo is in accordance with the colours of the clusters calculated by flowEMMi. The mean values and abundances of all cell clusters calculated by flowEMMi and FlowJo can be found in the additional file 026.csv. [file 12859_2019_3152_MOESM3_ESM.png]

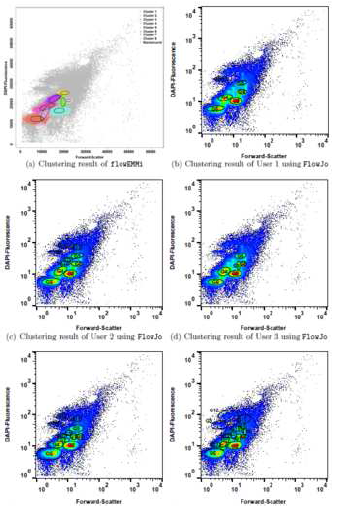

Supplement: Supplementary file 4 — Additional file 4 Clustering results for sample InTH_160715_019 using flowEMMi with 8 congruent cell clusters and 64.6% foreground cells (a) and manual clustering performed by 5 expert users using FlowJo (b-f). User 1 selected 6 cell clusters with 60.1% foreground cells (b). User 2 selected 10 cell clusters with 75.9% foreground cells (c). User 3 selected 6 cell clusters with 67.2% foreground cells (d). User 4 selected 12 cell clusters with 87.7% foreground cells (e). User 5 selected 15 cell clusters with 90.6% foreground cells (f). The label of the clusters selected by using FlowJo is in accordance with the colours of the clusters calculated by flowEMMi. The mean values and abundances of all cell clusters calculated by flowEMMi and FlowJo can be found in the additional file 019.csv. [file 12859_2019_3152_MOESM4_ESM.png]

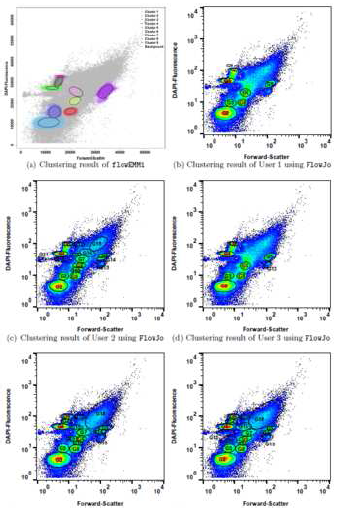

Supplement: Supplementary file 5 — Additional file 5 Clustering results for sample InTH_160714_033 using flowEMMi with 9 congruent cell clustersand 74.7% foreground cells (a) and manual clustering performed by 5 expert users using FlowJo (b-f). User 1 selected 7 cell clusters with 61.7% foreground cells (b). User 2 selected 17 cell clusters with 80.1% foreground cells (c). User 3 selected 8 cell clusters with 63.2% foreground cells (d). User 4 selected 16 cell clusters with 92.7% foreground cells (e). User 5 selected 17 cell clusters with 90.2% foreground cells (f). The label of the clusters selected by using FlowJo is in accordance with the colours of the clusters calculated by flowEMMi. The mean values and abundances of all cell clusters calculated by flowEMMi and FlowJo can be found in the additional file 033.csv. [file 12859_2019_3152_MOESM5_ESM.png]

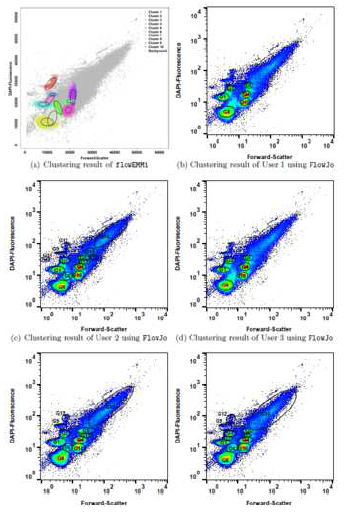

Supplement: Supplementary file 6 — Additional file 6 Clustering results for sample InTH_160729_027 using flowEMMi with 10 congruent cell clusters and 66.4% foreground cells (a) and manual clustering performed by 5 expert users using FlowJo (b-f). User 1 selected 6 cell clusters with 69.5% foreground cells (b). User 2 selected 14 cell clusters with 87% foreground cells (c). User 3 selected 6 cell clusters with 69.9% foreground cells (d). User 4 selected 11 cell clusters with 93.7% foreground cells (e). User 5 selected 12 cell clusters with 93% foreground cells (f). The label of the clusters selected by using FlowJo is in accordance with the colours of the clusters calculated by flowEMMi. The mean values and abundances of all cell clusters calculated by flowEMMi and FlowJo can be found in the additional file 027.csv. [file 12859_2019_3152_MOESM6_ESM.png]

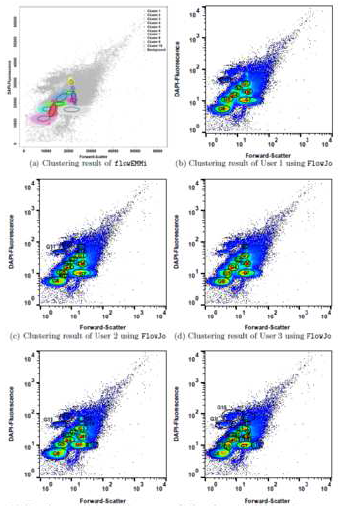

Supplement: Supplementary file 7 — Additional file 7 Clustering results for sample InTH_160715_020 using flowEMMi with 10 congruent cell clusters and 55.8% foreground cells (a) and manual clustering performed by 5 expert users using FlowJo (b-f). User 1 selected 8 cell clusters with 64.2% foreground cells (b). User 2 selected 13 cell clusters with 78.2% foreground cells (c). User 3 selected 8 cell clusters with 70.5% foreground cells (d). User 4 selected 13 cell clusters with 86.8% foreground cells (e). User 5 selected 17 cell clusters with 91.3% foreground cells (f). The label of the clusters selected by using FlowJo is in accordance with the colours of the clusters calculated by flowEMMi. The mean values and abundances of all cell clusters calculated by flowEMMi and FlowJo can be found in the additional file 020.csv. [file 12859_2019_3152_MOESM7_ESM.png]

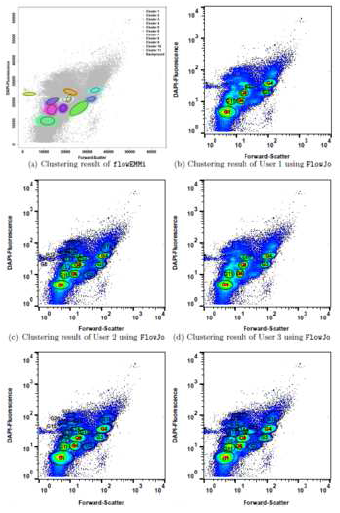

Supplement: Supplementary file 8 — Additional file 8 Clustering results for sample InTH_160720_035 using flowEMMi with 11 congruent cell clusters and 72.6% foreground cells (a) and manual clustering performed by 5 expert users using FlowJo (b-f). User 1 selected 7 cell clusters with 69.5% foreground cells (b). User 2 selected 17 cell clusters with 81.7% foreground cells (c). User 3 selected 7 cell clusters with 71.5% foreground cells (d). User 4 selected 17 cell clusters with 88.5% foreground cells (e). User 5 selected 15 cell clusters with 92% foreground cells (f). The label of the clusters selected by using FlowJo is in accordance with the colours of the clusters calculated by flowEMMi. The mean values and abundances of all cell clusters calculated by flowEMMi and FlowJo can be found in the additional file 035.csv. [file 12859_2019_3152_MOESM8_ESM.png]

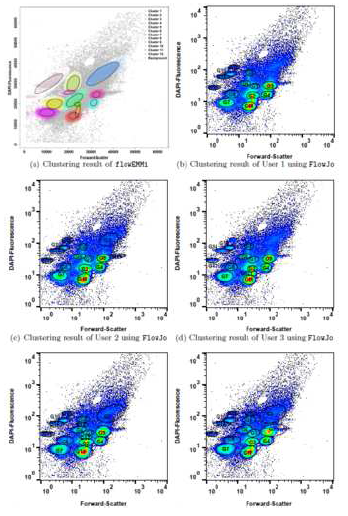

Supplement: Supplementary file 9 — Additional file 9 Clustering results for sample InTH_160712_025 using flowEMMi with 12 congruent cell clusters and 71.6% foreground cells (a) and manual clustering performed by 5 expert users using FlowJo (b-f). User 1 selected 13 cell clusters with 76.5% foreground cells (b). User 2 selected 15 cell clusters with 82.1% foreground cells (c). User 3 selected 13 cell clusters with 79.1% foreground cells (d). User 4 selected 16 cell clusters with 90.7% foreground cells (e). User 5 selected 15 cell clusters with 91.6% foreground cells (f). The label of the clusters selected by using FlowJo is in accordance with the colours of the clusters calculated by flowEMMi. The mean values and abundances of all cell clusters calculated by flowEMMi and FlowJo can be found in the additional file 025.csv. [file 12859_2019_3152_MOESM9_ESM.png]

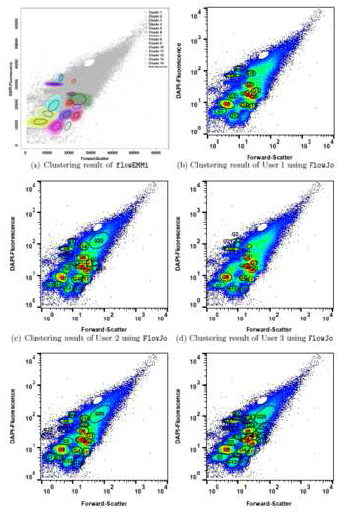

Supplement: Supplementary file 10 — Additional file 10 Clustering results for sample InTH_160713_012 using flowEMMi with 14 congruent cell clusters and 49.5% foreground cells (a) and manual clustering performed by 5 expert users using FlowJo (b-f). User 1 selected 13 cell clusters with 49.3% foreground cells (b). User 2 selected 20 cell clusters with 75.4% foreground cells (c). User 3 selected 14 cell clusters with 47.3% foreground cells (d). User 4 selected 19 cell clusters with 66.3% foreground cells (e). User 5 selected 25 cell clusters with 92% foreground cells (f). The label of the clusters selected by using FlowJo is in accordance with the colours of the clusters calculated by flowEMMi. The mean values and abundances of all cell clusters calculated by flowEMMi and FlowJo can be found in the additional file 012.csv. [file 12859_2019_3152_MOESM10_ESM.png]

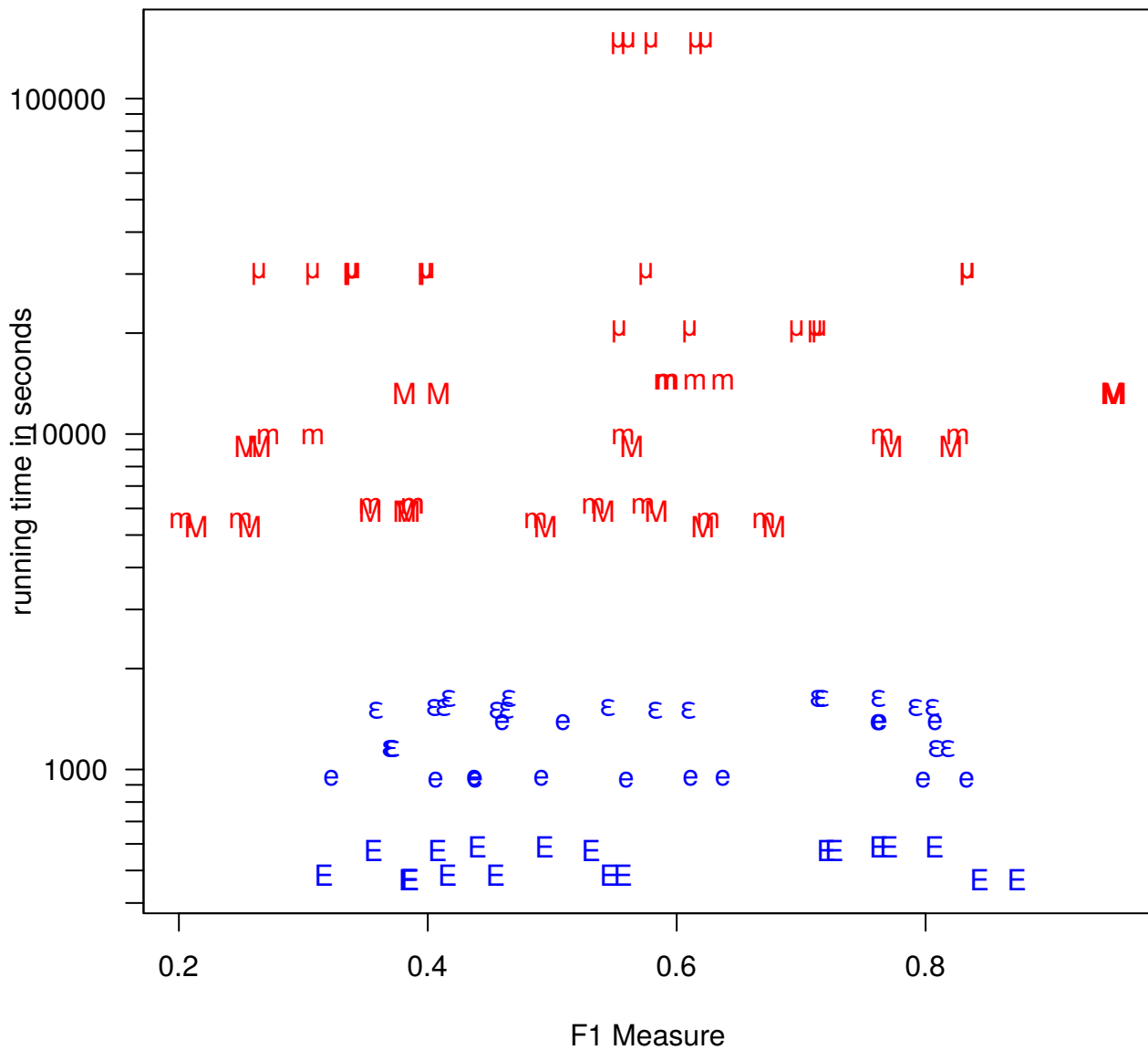

Supplement: Supplementary file 11 — Additional file 11 Comparison of running time (note the logarithmic scaling) vs F 1 score for flowEMMi (characters ε, e, Ein blue) and flowMerge(characters μ, m, M in red). flowEMMi yields, on average over all runs shown above ≈10.7% better F 1 scores (flowEMMi mean: 0.57, sd:0.17; flowMerge mean: 0.53, sd: 0.20), at very different time scales (flowEMMi mean: 1 012, sd: 427; flowMerge mean: 24 665, sd: 38 019). flowEMMi performs extremely well in a time constrained regime at early Expectation-Minimization cutoff (using on the cutoff at <1 instead of cutoff <0.01 or <10−5) with F 1 score mean: 0.56, sd:0.18, and a running time in seconds of mean: 528, sd: 53. While flowMerge has slightly worse F 1 score characteristics (mean: 0.54, sd: 0.24), with running times a lot higher (mean: 8 391, sd: 3 239). Since both algorithms are parallelized, actual wall-clock times are lower by a factor of 2–3 on a 4-core machine. Given running times are total core seconds used. [file 12859_2019_3152_MOESM11_ESM.pdf]

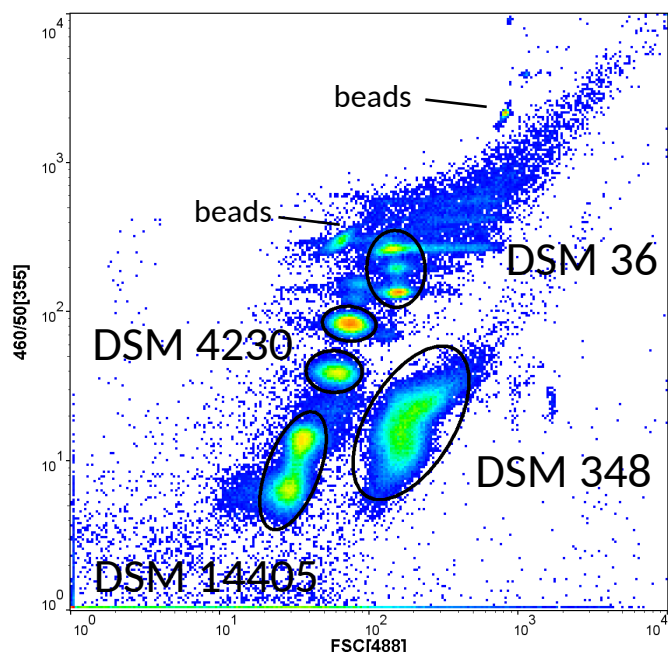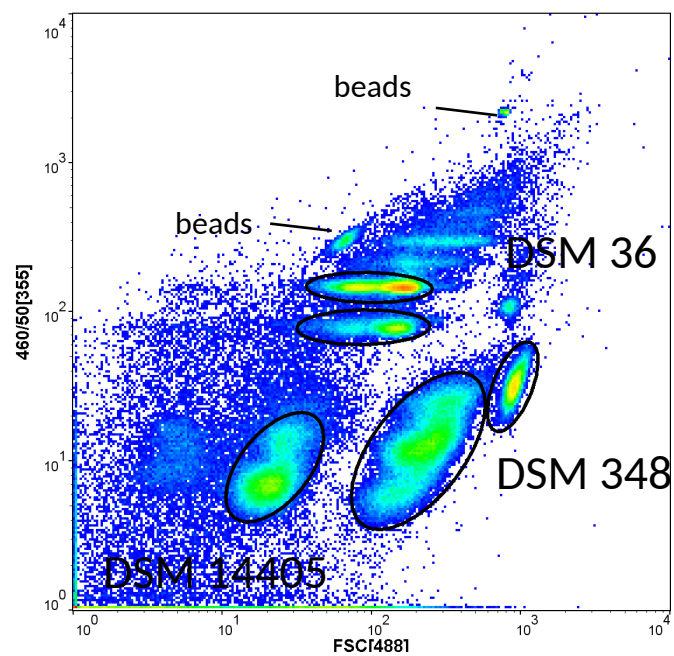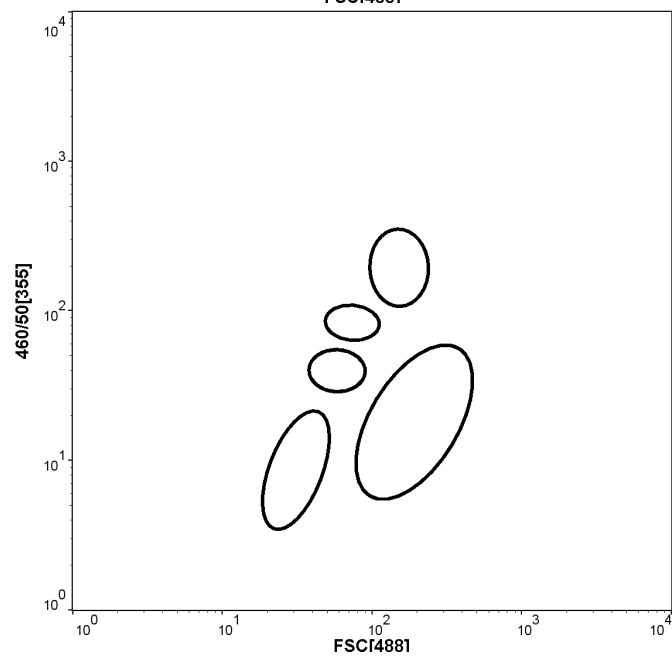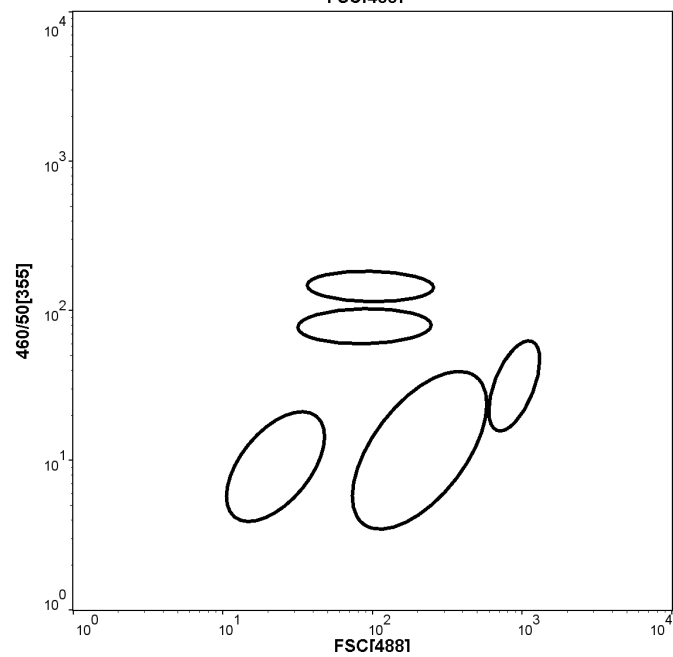

Supplement: Supplementary file 12 — Additional file 12 Flow cytometric measurement of microbial cytometric mock communities. Left: strains Stenotrophomonas rhizophila DSM 14405, Escherichia coli DSM 4230, Kocuria rhizophila DSM 348, and Paenibacillus polymyxa DSM 36 were grown in liquid culture, respectively. Right: strains Stenotrophomonas rhizophila DSM 14405, Kocuria rhizophila DSM 348, and Paenibacillus polymyxa DSM 36 were grown on plate. The beads were introduced for instrumental alignment of the flow cytometer. Below: manually set gate templates for the liquid (left) and plate (right) microbial cytometric mock communities. [file 12859_2019_3152_MOESM12_ESM.pdf]

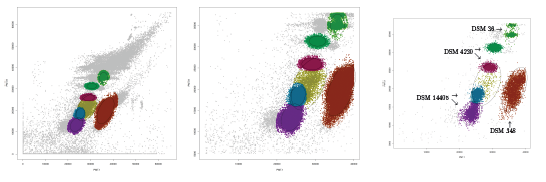

Supplement: Supplementary file 13 — Additional file 13 Automated gating by flowEMMi revealed the highest abundant subpopulations of the four strains Stenotrophomonas rhizophila DSM 14405, Escherichia coli DSM 4230, Kocuria rhizophila DSM 348, and Paenibacillus polymyxa DSM 36 grown in liquid culture. From left to right: full data set, including noise, rectangular cutout without corner noise, gating on subset of data. Automatic gating by flowEMMi yields an F 1 value of 0.85. [file 12859_2019_3152_MOESM13_ESM.png]

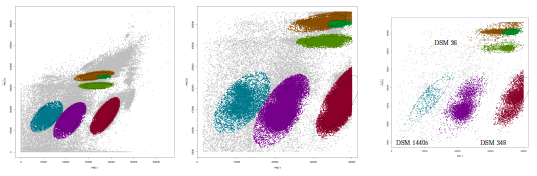

Supplement: Supplementary file 14 — Additional file 14 Automated gating by flowEMMi revealed the highest abundant subpopulations of the three strains Stenotrophomonas rhizophila DSM 14405, Kocuria rhizophila DSM 348, and Paenibacillus polymyxa DSM 36 grown on plate. From left to right: full data set, including noise, rectangular cutout without corner noise, gating on subset of data. Automatic gating by flowEMMi yields an F 1 value of 0.81. [file 12859_2019_3152_MOESM14_ESM.png]
